# Feasibility of an embedded palliative care clinic model for patients with an advanced thoracic malignancy

**DOI:** 10.1007/s00520-023-07621-w

**Published:** 2023-02-14

**Authors:** Erin M. Bertino, Madison M. Grogan, Jason A. Benedict, Julia L. Agne, Sarah Janse, Christine Eastep, Diana Sullivan, Kelly C. Gast, Michelle J. Naughton, Carolyn J. Presley

**Affiliations:** 1 Department of Internal Medicine, Division of Medical Oncology, The Ohio State University Comprehensive Cancer Center, Columbus, OH USA; 2 Center for Biostatistics, The Ohio State University Wexner Medical Center, Columbus, OH USA; 3 Department of Internal Medicine, Division of Palliative Medicine, The Ohio State University Wexner Medical Center, Columbus, OH USA; 4 Cancer Prevention and Control, The Ohio State University Comprehensive Cancer Center, Columbus, OH USA

**Keywords:** Feasibility, Thoracic malignancy, Palliative care, Embedded clinic, Oncology clinic

## Abstract

**Purpose:**

Early palliative care (PC) with standard oncology care has demonstrated improved patient outcomes, but multiple care delivery models are utilized. This study prospectively evaluated the feasibility of an embedded PC clinic model and collected patient-reported outcomes (PROs) and caregiver needs.

**Methods:**

In this observational study of embedded outpatient PC for patients with advanced thoracic malignancies treated at The Ohio State University Thoracic Oncology clinic, patients received same-day coordinated oncology and palliative care visits at one clinic location. PC encounters included comprehensive symptom assessment and management, advanced care planning, and goals of care discussion. Multiple study assessments were utilized. We describe the feasibility of evaluating PROs and caregiver needs in an embedded PC model.

**Results:**

Forty patients and 28 caregivers were enrolled. PROs were collected at baseline and follow-up visits. Over a 12-month follow-up, 36 patients discontinued study participation due to hospice enrollment, death, study withdrawal, or COVID restrictions. At baseline, 32 patients (80%) rated distress as moderate-severe with clinically significant depression (44%) and anxiety (36%). Survey completion rates significantly decreased over time: 3 months (24 eligible, 66% completed), 6 months (17 eligible; 41% completed), 9 months (9 eligible; 44% completed), and 12 months (4 eligible; 50% completed).

**Conclusion:**

We found that an embedded PC clinic was feasible, although there were challenges encountered in longitudinal collection of PROs due to high study attrition. Ongoing assessment and expansion of this embedded PC model will continue to identify strengths and challenges to improve patient and caregiver outcomes.

## Introduction

Thoracic malignancies are among the most common and deadly cancers, both in the USA and internationally [1]. Because of the high morbidity and mortality, patients with an advanced thoracic malignancy are at high risk for disease-related symptoms, including pain, loss of appetite, fatigue, cough, dyspnea, and depression/anxiety [2, 3]. Strategies to manage distressing symptoms may include treatment of the underlying advanced disease, such as systemic anti-cancer therapies or radiation, as well as symptom-directed management. Prior studies have demonstrated the importance of a multidisciplinary approach, including utilization of palliative medicine services early in the disease course to fully address symptom burden [4].

Multi-disciplinary oncologic care, including palliative care referral, is an American Society of Clinical Oncology clinical practice recommendation for lung cancer [5]. Despite this recommendation, multiple barriers to palliative care referral still exist, including oncologist or patient reluctance, specialist availability, and patient time burden [6, 7]. To address these issues, there are novel emerging models for delivering multidisciplinary palliative and oncology care. Three common care delivery models are free-standing palliative care clinics, co-located palliative care clinics, and embedded oncology-palliative (Onco-Pall) care clinics. Each clinic model has unique benefits and challenges to outpatient palliative care delivery [8]. The potential benefits of the embedded models include improved coordination to streamline care, as well as lower overall treatment burden including cost, parking, patient wait time, and travel time [4].

To improve palliative care access, The Ohio State University (OSU) Thoracic Oncology Clinic established an embedded Onco-Pall clinic in September 2018. To evaluate this practice model, we conducted a prospective evaluation of feasibility as well as patient- and caregiver-reported outcomes. We report here on this experience.

## Methods

### Study design and sample

**Fig. 1:**
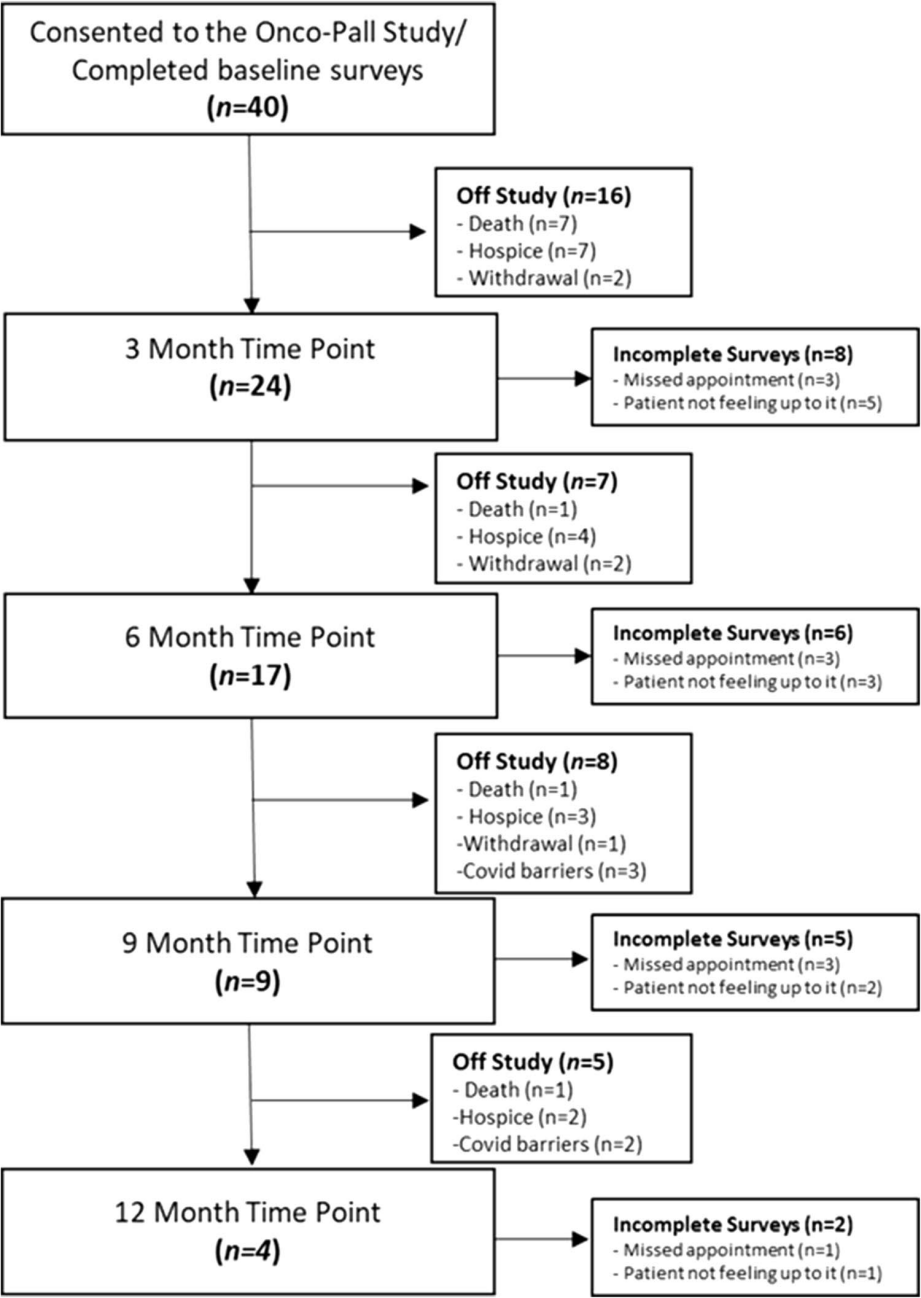
Study CONSORT diagram

Conducted between December 2018 and August 2020, this was a single-center, prospective pilot study of adult patients with either a new or established diagnosis of an advanced thoracic malignancy. All patients referred to Palliative Medicine from the Thoracic Oncology Clinic were offered study participation; those who enrolled in the study were evaluated by Palliative Medicine at the Onco-Pall embedded clinic. Patients that declined study enrollment but were interested in receiving palliative care were given the option to be seen at OSU’s free-standing palliative care outpatient clinic or through our Onco-Pall embedded model in the Thoracic Oncology clinic. The Onco-Pall embedded clinic included a single-physician clinic available 2 days per week in The Ohio State University Thoracic Medical Oncology clinic. The clinic workflow is described in detail below (see Fig. 1 for CONSORT diagram).

#### Patient selection

Eligible patients included any adult patient (at least 18 years old) with advanced or metastatic cancer of the chest (lung, thymus, or pleura) receiving treatment at The Ohio State University Thoracic Oncology clinic. Both new and established oncology patients were included. New patients were defined as those who either were treatment naïve or had started treatment for metastatic disease within the last 30 days. Established patients were all patients with advanced cancer receiving anti-cancer therapy for > 30 days prior to study enrollment. There was no limit on prior therapies. Patients who were already established with outpatient palliative medicine or enrolled in hospice were excluded. Inpatient palliative care consultation prior to study enrollment was permitted.

#### Caregiver selection

Patients were asked to identify a caregiver to participate in the trial although this was not required for patient participation. Caregivers included any support person involved in the patient’s care (including spouse, partner, child, and friend).

### Study intervention

This study was reviewed and approved by The Ohio State University Institutional Review Board prior to patient enrollment. Patients and caregivers provided informed consent prior to study participation.

The primary intervention was referral for palliative care assessment in the Onco-Pall embedded palliative care clinic. Patients were evaluated by the palliative care physician in the Thoracic Oncology Clinic and were given information regarding the study. Patients expressing interest met with the study clinic research coordinator to undergo the informed consent process. The palliative care physician evaluation was tailored to individual patient needs, but included symptom management (physical and psychological), advanced care planning (advanced directives and goals of care discussion), caregiver support, and referral for adjunctive supportive services as needed (including dietician, chaplain, social work, psychology). To assess symptoms, patients and caregivers completed study assessments as described below (see study assessments).

The addition of a palliative care physician in the Thoracic Oncology Clinic also involved standardization of controlled substance prescribing practices per best-practice guidelines [9]. A controlled substance agreement was completed by all patients receiving controlled substance prescriptions. Per institutional practice standards, all patients receiving controlled substance prescriptions were required to provide urine drug screens (UDS) at the initial visit and at least every 6 months thereafter to monitor safety and compliance with controlled substances. UDS results were analyzed using both immunoassay and confirmatory chromatography-based analysis through Dominion Diagnostics, LLC, an accredited national medical laboratory and reviewed by a clinical pharmacist as previously described by Kumar et al. [10].

### Onco-Pall clinic workflow

The Thoracic Oncology Clinic includes up to 12 exam rooms (four rooms per medical oncology attending physician) with an adjacent infusion suite with 19 infusion chairs. Each medical oncology attending physician is supported by one or two nurses, depending on patient volume, and shares a patient care associate (PCA) with another attending physician. The Thoracic Oncology Clinic also has a dedicated medical oncology phone triage nurse and clinical pharmacist (PharmD). The on-site palliative medicine physician received support from one dedicated nurse and a shared PCA. Palliative medicine also has a shared off-site clinical pharmacist and designated phone triage nurse for palliative providers. A nurse case manager and social worker for the Thoracic Oncology team were also available to assist the palliative medicine physician with patients in the Onco-Pall embedded clinic. Medical oncology providers (physicians or advanced practice providers (APPs)) placed palliative medicine referrals per standard practice depending on patient needs. Referrals indicated preference for the Onco-Pall clinic. Patients were then scheduled for same-day visits with medical oncology and palliative medicine. The palliative medicine physician maintained an independent patient schedule with 30-min return visits and 60-minute new patient visits.

On the day of the visit, patients were evaluated either during a joint visit or sequential visits, depending on patient needs. Separate nursing assessments were performed by oncology and palliative nurses at check-in. Joint visits were generally conducted for complex symptom management or goals of care discussions. Sequential visits by the palliative medicine team could be conducted either in oncology clinic exam rooms or in an adjacent chemotherapy infusion suite depending on patient schedule. Patient care questions were coordinated by direct physician-to-physician communication in the team workroom as palliative medicine shares the same workspace with medical oncology.

Concerning clinical capacity, the palliative medicine physician maintained an independent clinic schedule. The number of shared versus sequential provider visits was not tracked.

### Outcomes

The primary outcome was feasibility of the embedded, palliative care model including perceived demand (palliative care referral rate), clinician capacity (scheduling access for embedded visits), resource assessment (clinic space, support staff utilization), and collection of patient- and caregiver-reported outcomes. Referral rate was defined as the total number of palliative medicine referrals placed during the study period. The time to referral and changes in referral patterns are reported separately [6]. Secondary outcomes evaluated study adherence and patient acceptability of the care model.

### Study assessments

#### Patient/caregiver assessments

Patients completed surveys to evaluate symptoms including depression, anxiety, and functional status. The National Comprehensive Cancer Network (NCCN) Distress Thermometer and Problem List and Functional Activity Scale (FAS) [11–14] surveys were collected at each visit (every 3-to-4 weeks). Patients were also asked to complete the following surveys every 3 months for up to 1 year: European Organization for Research and Treatment of Cancer (EORTC), Quality of Life of Cancer Patients (QLC-C30), Quality of Life-Lung Cancer 13 (QLQ-LC-13) [15, 16], and the Hospital Anxiety and Depression Scale (HADS) [17]. For EORTC, the scores range from 0 to 100 with higher scores indicating a healthy level of functioning and lower scores indicating a high level of symptomatology. The HADS is a 14-item scale with seven items each for anxiety and depression subscales. Scoring for each item ranges from 0 to 3. A subscale score ≥ 8 denotes anxiety or depression [18]. At baseline only, patients completed the Screener and Opioid Assessment for Patients with Pain-Revised (SOAPP-R) to assess opioid abuse risk [10, 19]. Patient acceptability questions regarding patient opinions within the Onco-Pall model were completed once at the 3-month time point. This consisted of five questions to assess patient preferences regarding palliative care appointments, including timing, location, length of visit, and open-ended feedback. The caregivers were asked to complete the Supportive Care Needs Survey–Patients & Caregivers (SCNC-P&C) at baseline and then every 3 months for up to 1 year [20, 21].

### Statistical analysis

Descriptive statistics were used to summarize demographic information with totals and percentages used for categorical data and means and standard deviations used for continuous data. Percentage data were analyzed and reported for each section of the questionnaires and summarized in figures sorted by issues of least concern to most concern. Although patients may have completed the survey at multiple time points, only the baseline responses are displayed as too few patients completed subsequent surveys to allow for longitudinal analyses. All analyses and graphs were generated in R version 4.1.

## Results

A total of 40 patients were enrolled between December 2018 and September 2019. Patient demographics are detailed in Table 1. The study enrolled both newly diagnosed (32.5%) and established patients (67.5%). The median age at baseline was 60 years (range 30–80 years) and more than half of the patients were male (60%). Of the study participants, 38 were Caucasian and two were Black/African American. Non-small cell lung cancer (NSCLC) was the most common diagnosis (33 patients, 82.5%). As expected, most patients who enrolled had stage IV metastatic disease at diagnosis (85%). There was a significant increase in palliative medicine referrals from thoracic oncology after the embedded palliative medicine physician joined the Onco-Pall clinic. In 2017–2018, 65 referrals were placed. From 2018 to 2019, palliative medicine referrals increased to 160 (146% increase). Physician-perceived barriers to palliative care were also assessed and previously described [6]. Briefly, these barriers included time burden to patients, patient preference, and location of free-standing palliative care clinic.

**Table 1:** Baseline patient demographics and characteristics (*n* = 40)

Characteristic	*n* (%)
Age	
≤ 65 years	23 (57.5)
66–69	8 (20.0)
70–74	5 (12.5)
75–79	3 (7.5)
≥ 80	1 (2.5)
Sex	
Male	24 (60.0)
Female	16 (40.0)
Race	
Black or African American	2 (5.0)
White	38 (95.0)
Ethnicity	
Hispanic or Latino	1 (2.5)
Not Hispanic or Latino	39 (97.5)
Cancer type	
NSCLC	33 (82.5)
SCLC	7 (17.5)
Histology	
Adenocarcinoma	19 (47.5)
Squamous	9 (22.5)
Adenosquamous	2 (5.0)
Large cell	2 (5.0)
NSCLC NOS	1 (2.5)
SCLC	7 (17.5)
Stage at diagnosis	
IA	1 (2.5)
IB	1 (2.5)
IIIA	2 (5.0)
IIIB	2 (5.0)
IV	34 (85.0)
Body mass index	
< 18.5	3 (7.5)
18.5–24.9	11 (27.5)
25–29.9	13 (32.5)
≥ 30	13 (32.5)

### Patient-related outcomes

**Fig. 2:**
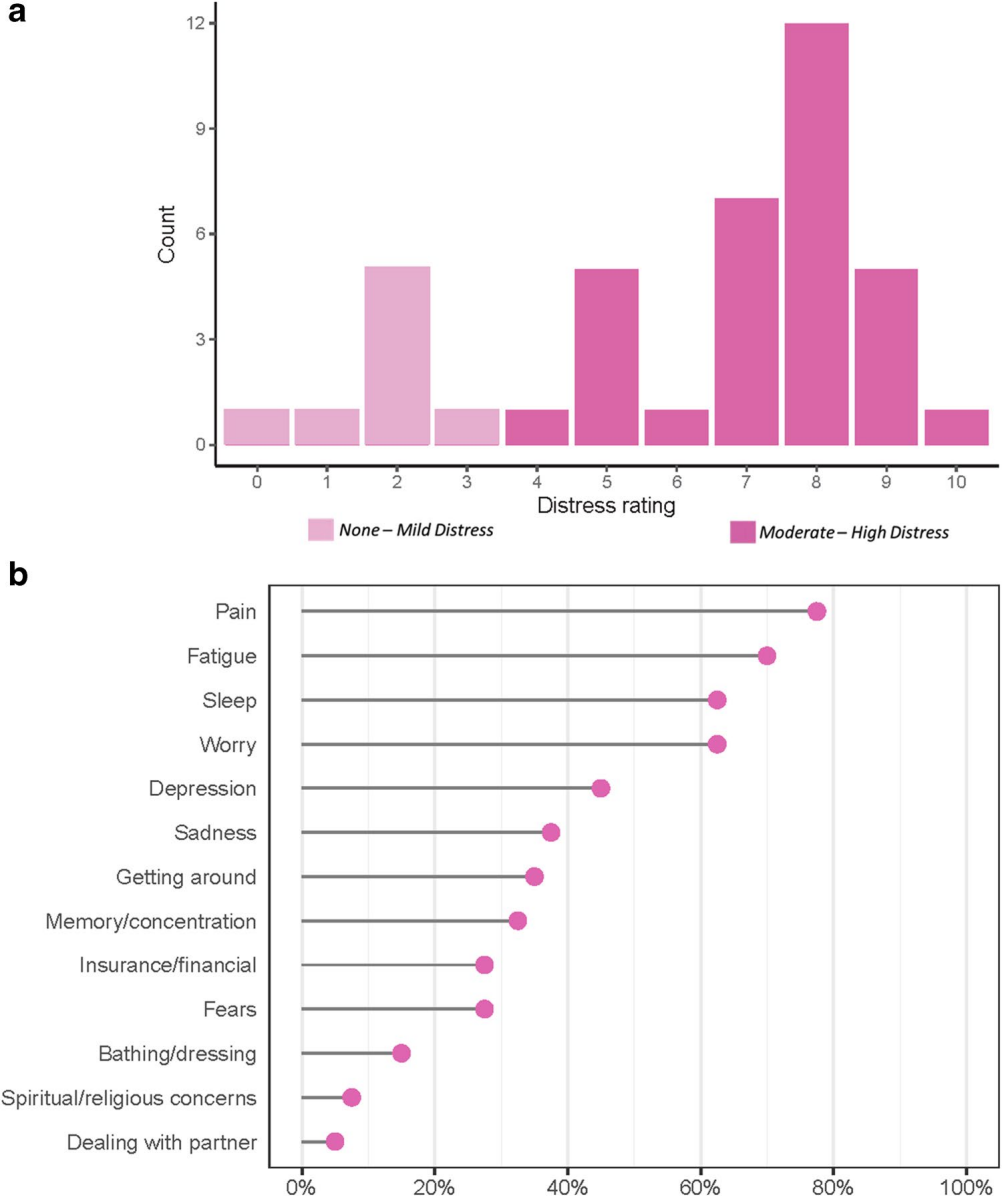
**a** National Comprehensive Cancer Network (NCCN) distribution at baseline for patients. **b** National Comprehensive Cancer Network (NCCN) Problem List identified at baseline for patients

**Fig. 3:**
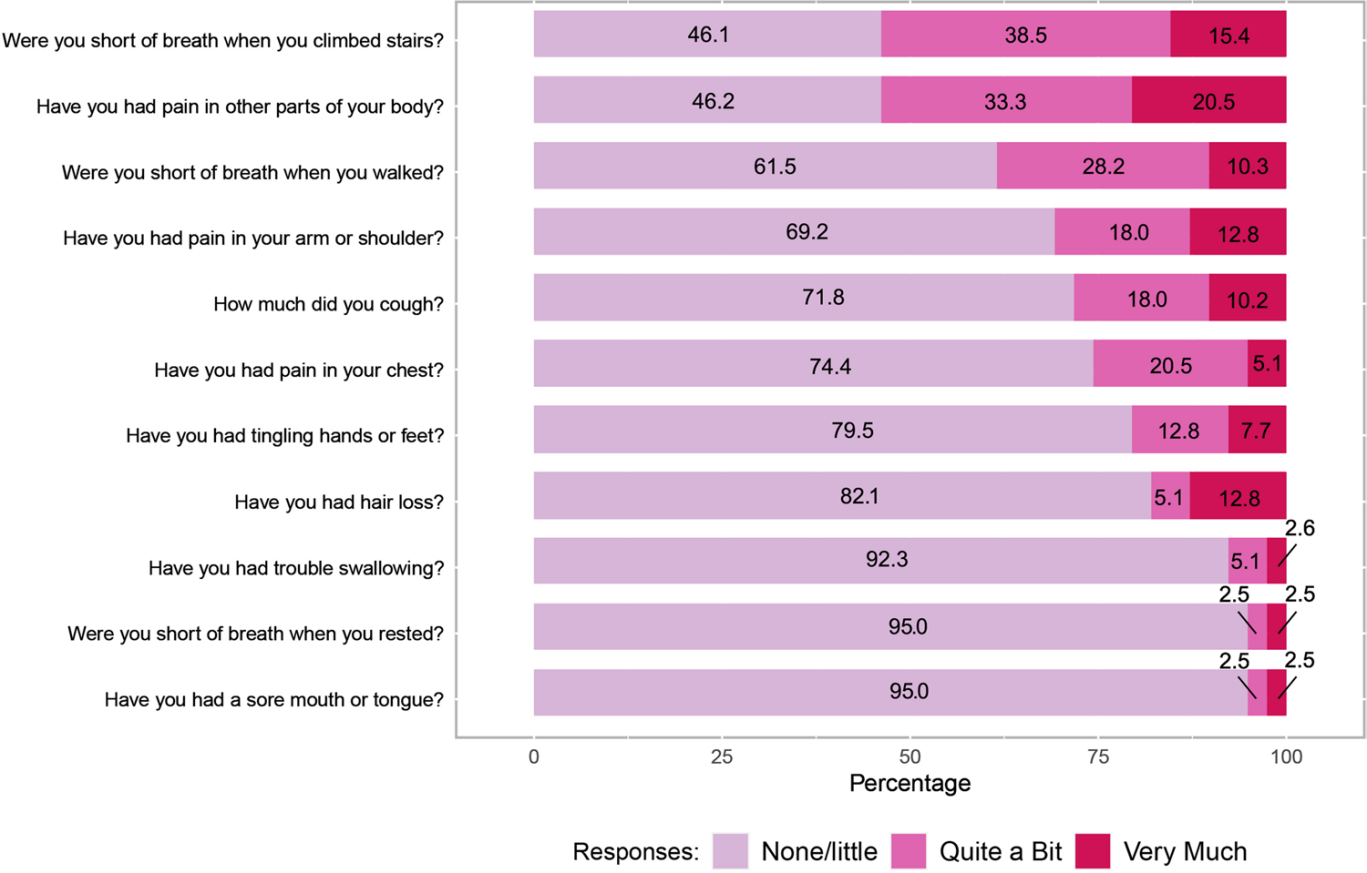
EORTC-lung cancer supplement (*LC-13) identified at baseline for patients. EORTC, European Organization for Research and Treatment of Cancer; LC-13, Lung cancer-13 item supplement

At baseline, most patients (*n*=24, 80%) rated their distress level as moderate-high (score ≥ 4) on the NCCN Distress Thermometer (Table 2 and Fig. 2a). This included 15 patients with baseline distress scores ≥ 8 (severe/extreme distress). For the NCCN Problem List, the most common concerns were pain (78%), fatigue (70%), sleep (62%), and worry (62%) (Fig. 2b). In contrast, social and practical concerns such as insurance/financial concerns (28%) and spiritual/religious concerns (7.5%) were rated lower among our patient population. The baseline median FSA score was 3, indicating a low level of disability among the patients (range 0–26, with higher scores associated with increased disability) (Table 2). The median EORTC global health score at baseline was 50 (range 0–83). For the LC-13 component, the mean score was 26.8 (SD 12.3) with cough, exertional dyspnea, sore throat, and pain (chest as well as other) as the most common symptoms (Fig. 3). For HADS, the mean score for depression was 6.7 (SD 4.4) and anxiety was 6.9 (SD 4.5); 17 patients had scores ≥ 8 indicating significant depression symptoms, and 14 patients had significant anxiety (scores >8) (Table 2).

**Table 2:** Patient-reported outcomes at baseline (*n* = 40)

Patient-reported outcome at baseline	Completed (*n*)	*n* (%)
NCCN distress thermometer	40	
0–3 (no distress – low distress)		8 (20.0)
4–10 (moderate – high distress)		32 (80.0)
Functional Activity Scale	40	
13 Item score (ADLs + IADLS)		
0–2		20 (50.0)
3–6		11 (27.5)
7–13		9 (22.5)
7 Item score (ADLs)		
0		30 (75.0)
1		7 (17.5)
2		2 (5.0)
≥ 3		1 (2.5)
EORTC--Quality of Life Questionnaire, mean (SD)	39	
Symptom score (range 0–100)		36.4 (17.4)
Functional score (range 0–100)		46.7 (37.6)
Global Health score (range 0–100)		47.9 (21.2)
EORTC – Lung Cancer Supplement score (range 0–100)		26.8 (12.3)
Hospital Anxiety and Depression Scale	39	
Depression score total, mean (SD)		6.7 (4.4)
Depression score ≥ 8		17 (43.6)
Anxiety score total, mean (SD)		6.9 (4.5)
Anxiety score ≥ 8		14 (35.9)

*NCCN*, National Comprehensive Cancer Network; *EORTC*, European Organization for Research and Treatment of Cancer

Notes: One patient did not complete both the EORTC-Quality of Life Questionnaire and the Hospital Anxiety and Depression Scale. A score ≥ 8 on Hospital Anxiety and Depression Scale indicates clinically significant anxiety and/or depression and was found to best correlate with the Montgomery-Asberg Depression Rating Scale (MADRS) [17]

#### Study attrition

The attrition rate for study follow-up was significant (90%), particularly in the first 6 months after enrollment. In the first 3 months, only 60% of patients completed the 3-month follow-up (i.e., 7 patients died, 7 enrolled in hospice, and 2 withdrew consent). At 6 months, 7 out of 17 eligible patients completed surveys (41%). Hospice referrals and patient deaths were again the most common reasons for non-completion. At 9 and 12 months, 44% and 50% of eligible patients, respectively, were able to complete the surveys. The acceptability questions at 3 months were completed by 16 patients, with results indicating that patients preferred the current care model. Patients also gave feedback regarding surveys and requested “less questions.” One patient felt visits were too long.

The study CONSORT diagram (Fig. 1) details enrollment and study follow-up. The most common reason for patient withdrawal over the course of the study was either death (27.8%) or hospice transition (44.4%). Of the patients who remained enrolled, patients’ refusal to complete surveys or missed appointments were the most common reasons for missing survey data. For the patients who remained in the study and were able to complete assessments, results demonstrated no change in levels of distress by the NCCN Distress Thermometer, although the attrition rate in survey completion was high (52% completion rate by 3 months, 25% completion rate by 6 months, 10% at 9 months, and 5% at 12 months).

### Caregiver outcomes

**Fig. 4:**
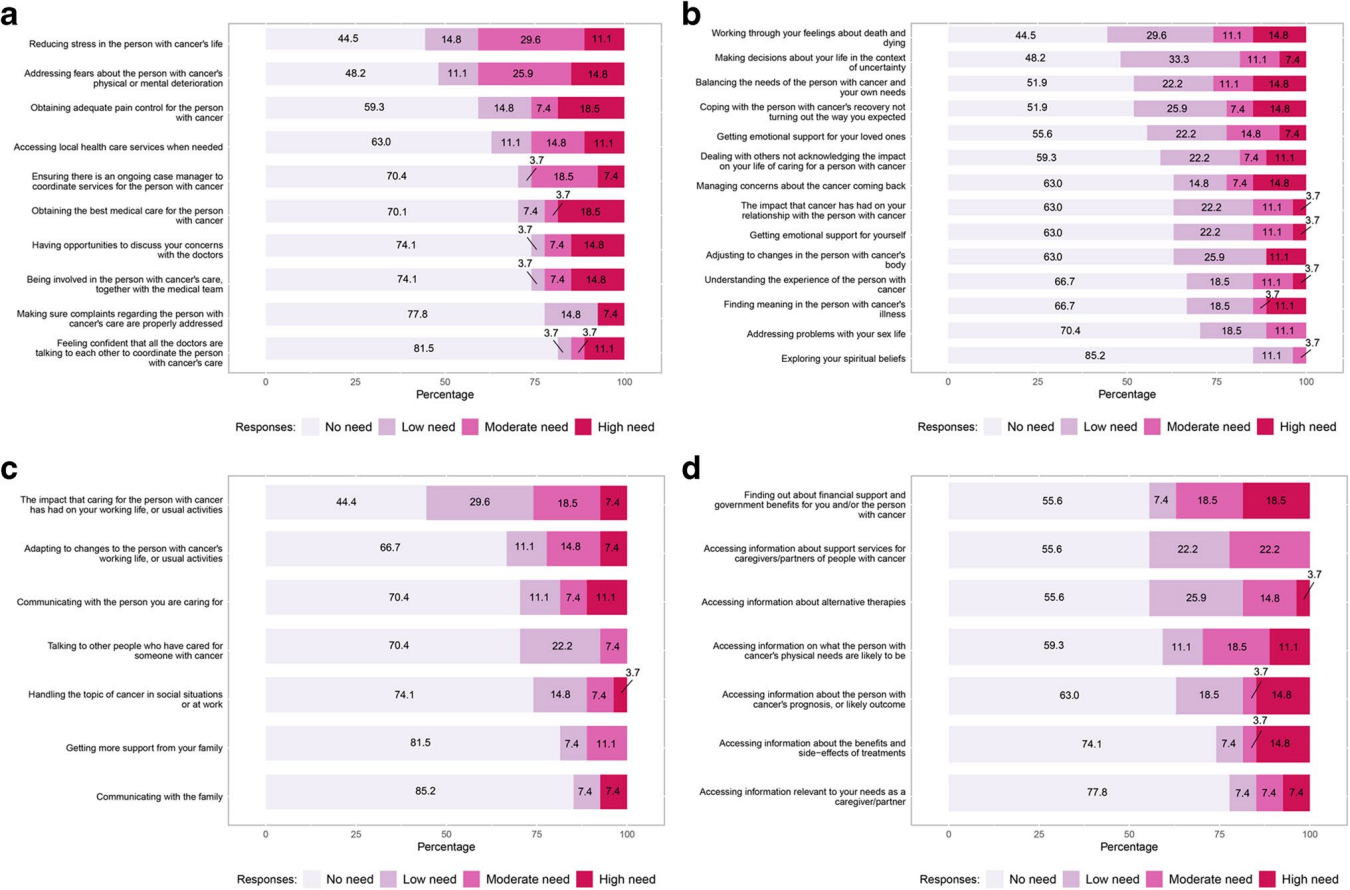
Caregiver domains of need at baseline. **a** Healthcare service needs. **b** Psychological and emotional needs. **c** Work and social needs. **d** Information needs

Caregiver outcomes (*n*=28) are summarized in Fig. 4a–d. All caregivers completed baseline assessments, but only 13 completed follow-up surveys (i.e., 13 at 3 months, 3 at 6 months, 1 at 9 months). Barriers to follow-up included inconsistent attendance at appointments and caregivers declining to participate in subsequent visits. The caregiver survey assessed four domains for underlying needs of caregivers: healthcare service needs, psychological and emotional needs, work and social needs, and information needs [20]. The most common high-need area identified was healthcare service needs, particularly pain management, reducing stress for patients, and caregiver fears about patient decline (Fig. 4a). For psychological and emotional needs, there were multiple areas of moderate-to-high need including balancing caregiver and patient needs, addressing feelings about death and dying, and concerns about potential cancer recurrence (Fig. 4b). For work and social needs, the area of highest need was impact on work or usual activities for the caregiver (Fig. 4c). Additional areas of need were communication with the patient and adapting to changes in usual activities. Areas of high need in information included prognostic and outcome-related information, as well as understanding treatment risks and benefits. In addition, caregivers wanted information about financial support, insurance benefits, and future physical needs of the patient (Fig. 4d).

## Discussion

The key role of palliative care in the management of advanced lung cancer has been repeatedly demonstrated, yet the optimal model for providing this care has not yet been defined. This study further describes the workflow of an embedded model in contrast to other common care delivery models including free-standing and co-located palliative care clinics. Our distinctive model allowed for direct communication between palliative care and oncology physicians to facilitate timely communication and care transitions. This model also allowed opportunities for joint visits, which were particularly valuable for patients with complex symptoms or end-of-life care transitions. Overall, we found that palliative referrals increased with improved patient access, and we suspect this was largely due to a lowered appointment burden that our embedded model offers, including cost, parking, patient wait time, and travel time. While longitudinal symptom assessment was challenging among our patient population, we found valuable insights on the optimal modality for patient-reported outcome collection. In addition, we gained an understanding of the most common caregiver needs and of current gaps in the management of mental health concerns among caregivers.

Previous literature has shown that early palliative care, in both embedded and standalone clinics, has the potential to decrease depressive symptoms, improve quality-of-life measures, improve survival, and decrease aggressive end-of-life care [4, 22, 23]. Our embedded model had several shared features with pre-existing, palliative care clinics, as well as some that were unique. We suspect the in-person physician model with physicians co-located in the same workroom allowed for significantly improved face-to-face physician coordination and teamwork. We hypothesize this close interaction was likely the major factor in increasing palliative referrals and acceptability among the Thoracic Oncology clinic. Additionally, this finding suggests the decreased treatment burden among embedded models is a motivator for not only the placement of palliative referrals, but also patient completion of referrals. Furthermore, the embedded palliative model should be considered as an optimal format for early integration of palliative care among Thoracic Oncology clinics, as it has the potential to significantly decrease emergency department visits and hospitalizations and expand access, further described in Agne et al. and Gast et al. [6, 24].

Our embedded model only allowed for a palliative physician on two of the five clinic days due to limited resource availability. These findings suggest the implementation of a palliative physician on all 5 days could provide further benefit, increasing the capacity for early palliative intervention among our entire patient population. Additionally, patient-reported outcome collection should be designed so patients can choose to remotely complete questionnaires at a time best-suited for individual needs. Patient acceptability indicated a preference for shorter surveys at less frequent timepoints. Both patient refusals to complete all surveys and the direct patient feedback indicated that patients had “survey fatigue” and were unable/unwilling to complete multiple symptom surveys. For our population, we noted that in-person, patient-reported outcome assessments were more effective than email/electronic surveys, particularly if they were experiencing high symptom burden. Previous findings indicate that electronic symptom management may lead to improved symptom management, suggesting a more simplified electronic patient-reported outcome platform may be an effective alternative to receive more comprehensive survey data. Additionally, patients not familiar with technological platforms could benefit from a one-on-one training session to troubleshoot and improve patient comprehension and subsequent electronic-survey adherence.

This study has laid the foundation for an upcoming quality improvement project designed to implement a novel patient-facing tool, “MyPROfile-TC: Patient- and Caregiver-Reported Outcomes (PRO) & Symptom Monitoring with Triggered Referral Pathways for Thoracic Cancers,” in which patients will be able to select and answer questionnaires that are applicable to individual concerns. Additionally, MyPROfile-TC will implement cutoff scores and response flags to alert differing, individualized first points of contact based on patient responses. Communications will then follow a specific provider flow, depending on the measure, for the appropriate routes of care management. This will be a valuable tool tailored for patients with a thoracic cancer and their caregivers, with the goal of proactive, timelier responses to patient and caregiver concerns, improving quality of life with the eventual goal of decreasing the need for acute or emergent healthcare such as emergency department visits and hospitalizations. Optimizing an embedded Onco-Pall thoracic clinic using this patient-centered approach and tailoring to patients’ needs and preferences is an area of future research.

One limitation of this data was the significant drop-out rate over the 12-month study period. Our baseline patient-reported outcome results indicate that attrition was related to high symptom burden, hospice referral, and death, which suggests that using a shorter time frame in collecting patient-reported outcomes from patients may be needed for most patients. For future assessments, focusing on newly diagnosed patients or those earlier in their disease course, ideally within the first 2 months of diagnosis, may provide more insight on patient-related outcomes due to increased follow-up time. Another limitation of this trial was the limited caregiver data including absence of demographics and mental health evaluations. While other studies have performed depression/anxiety assessments in caregivers, we did not include this in our pilot; it is an area of future investigation. Ultimately, our results suggest caregivers may benefit from additional support services such as psychology/counseling, caregiver support groups, or access to services such as transportation. Targeted interventions designed to improve caregiver needs and outcomes could provide great benefit to those caring for someone diagnosed with an advanced thoracic malignancy. Despite these limitations, this feasibility study provided rationale and outcomes data, which allowed us to obtain a full-time palliative care APP within the Thoracic Oncology clinic.

In conclusion, we determined the embedded palliative care workflow was well-accepted and feasible in our Thoracic Oncology Clinic for providers, nursing, and patients. Referrals increased with associated improvement in patient access, but patient symptom assessment longitudinally was challenging. We plan to continue to evaluate models to improve palliative care access and improve patient and caregiver outcomes through the launch of MyPROfile-TC, specifically designed for patients and caregivers with advanced thoracic malignancies.

## Data Availability

Not applicable.
